# Prenatal Activation of Toll-Like Receptor-4 Dampens Adult Hippocampal Neurogenesis in An IL-6 Dependent Manner

**DOI:** 10.3389/fncel.2016.00173

**Published:** 2016-06-30

**Authors:** Abdeslam Mouihate

**Affiliations:** Department of Physiology, Faculty of Medicine, Health Sciences Centre, Kuwait UniversityKuwait City, Kuwait

**Keywords:** cytokines, RU486, doublecortin, Tbr2, perinatal

## Abstract

Prenatal immune challenge has been associated with alteration in brain development and plasticity that last into adulthood. We have previously shown that prenatal activation of toll-like receptor 4 by lipopolysaccharide (LPS) induces IL-6-dependent STAT-3 signaling pathway in the fetal brain. Whether this IL-6-dependent activation of fetal brain results in long lasting impact in brain plasticity is still unknown. Furthermore, it has been shown that prenatal LPS heightens the hypothalamic–pituitary–adrenal (HPA) response in adulthood. In the present study we tested whether LPS administration during pregnancy affects neurogenesis in adult male offspring. Because corticosterone, the end-product of HPA axis activity in rats, alters neurogenesis we tested whether this enhanced HPA axis responsiveness in adult male offspring played a role in the long lasting impact of LPS on neurogenesis during adulthood. Pregnant rats were given either LPS, or LPS and an IL-6 neutralizing antibody (IL-6Ab). The newly born neurons were monitored in the subventricular zone (SVZ) and the dentate gyrus (DG) of the hippocampus of adult male offspring by monitoring doublecortin and T-box brain protein-2 expression: two well-established markers of newly born neurons. Prenatal LPS decreased the number of newly born neurons in the DG, but not in the SVZ of adult offspring. This decreased number of newly born neurons in the DG was absent when IL-6Ab was co-injected with LPS during pregnancy. Furthermore, administration of a corticosterone receptor blocker, RU-486, to adult offspring blunted the prenatal LPS induced decrease in newly born neurons in the DG. These data suggest that maternally triggered IL-6 plays a crucial role in the long lasting impact of LPS on adult neurogenesis.

## Introduction

Birth of new neurons (neurogenesis) persists into adulthood in two discrete areas of the adult brain, the subventricular zone (SVZ) and dentate gyrus (DG) of the hippocampus. The newly born neurons functionally integrate into neuronal networks and have been shown to play a role in cognitive functions such as learning and memory (Lemaire et al., 2000; Abrous et al., 2005; Glasper et al., 2012; Gheusi et al., 2013; Lepousez et al., 2013; Khan et al., 2014). Several studies have shown that prenatal immune activation with lipopolysaccharide (LPS); the outer coat of gram negative bacteria, dampens neurogenesis in adult rodents (Cui et al., 2009; Graciarena et al., 2010, 2013; Lin and Wang, 2014), induces a deficit in their cognitive functions (Graciarena et al., 2010) and enhances stress response and depression-like behavior (French et al., 2013; Lin and Wang, 2014). The mechanism underlying such long lasting impact of LPS on neurogenesis is not clear yet.

It has been shown that LPS does not cross the feto-placental barrier but it rather exerts its effect through inducible pro-inflammatory cytokines (Ashdown et al., 2006). Indeed, LPS activates toll-like receptor-4 (TLR4) expressed in maternal macrophages, monocytes and Kupffer cells (Mouihate et al., 2008). LPS-binding to TLR4 activates the nuclear factor-κB (NFκB) which translocates into the nucleus and promotes the synthesis and release of a set of pro-inflammatory cytokines such as tumor necrosis factor (TNFα), interleukin-1β (IL-1β), and interleukin-6 (IL-6; Boksa, 2010). Experimental evidence suggests that IL-6, but not TNFα or IL-1β, crosses the placenta (Gayle et al., 2004; Dahlgren et al., 2006) and could directly affect fetal brains (Mouihate and Mehdawi, 2016). Therefore, we hypothesized that maternal immune challenge with LPS may alter neurogenesis in adult offspring in an IL-6 dependent manner. Experimental evidence suggests that perinatal exposure to immune challenge heightens the responsiveness of the hypothalamic–pituitary–adrenal (HPA) to either immune or psychological stresses, leading to increased levels of corticosterone, the end product of HPA activity (Mouihate et al., 2010; Enayati et al., 2012; Lin et al., 2012; Mouihate, 2012; Zager et al., 2014). Because corticosterone dampens neurogenesis (Schoenfeld and Gould, 2013; Chetty et al., 2014; Opendak and Gould, 2015), we also explored whether this hormone plays a role in the impact of prenatal immune stress on adult neurogenesis.

## Materials and Methods

### Animal Treatment

Male and female Sprague Dawley rats were maintained at 22°C on 12 h light/dark cycle (7 AM–7 PM), where food and water were available *ad libitum.* Female rats were mated with proven breeder male rats and vaginal smear were monitored daily for signs of copulation. Positively identified females with vaginal deposition of sperm were separated from the males and housed individually. On gestation day 15 (GD15), pregnant rats received intraperitoneal injections of either LPS (*Escherichia coli* O26:B6, Sigma–Aldrich, St. Louis, MO, USA) or saline in the presence or the absence of an IL-6 neutralizing antibody (IL-6Ab, 10 μg/kg i.p., from R&D systems, Inc., Minneapolis, MN, USA). The IL-6Ab is an IgG polyclonal antibody made in goat and purified by rat IL-6 affinity chromatography. We have previously shown that this antibody has no pyrogenic activity and that goat serum *per se* does not impact LPS-activated STAT-3 signaling pathway in the fetal brain (Mouihate and Mehdawi, 2016). Pregnant rats were randomly assigned to one of the following four groups. Rat group 1 received IL-6Ab followed 2 h later with LPS (100 μg/kg, i.p.), rat group 2 received IL-6Ab followed 2 h later with pyrogen-free saline, rat group 3 received pyrogen-free saline 2 h before injection of LPS (100 μg/kg), rat group 4 received two injections of pyrogen-free saline in 2 h apart. All injections were performed on the morning between 09:00 and 11:00. IL-6Ab was injected 2 h before LPS injection to allow for the IL-6 neutralizing action before the immune insult as previously described (Rummel et al., 2006; Mouihate and Mehdawi, 2016). Each rat group includes at least five pregnant dams. From each dam only one male rat was randomly selected for each of the rat group. These experiments were performed over a period of 2 years and include data from three mating at different times of the year. All experiments were done in accordance with the guidelines on humane handling of experimental animals as established by the Canadian Council on Animal Care. The procedures employed were approved by the ethical committee at Animal Resources Centre of Kuwait University.

Pregnant rats gave birth to normal size litters. Pups were weaned when they reach the age of 21 days. Male rats were group-housed as four animals per cage until they were 45 days old. After this time, animals were housed two per cage for the remainder of the experiment. This study included only male offspring because ovarian hormones affect neurogenesis and the magnitude of neurogenesis varies as a function of the phase of the estrous cycle in female offspring (Tanapat et al., 1999, 2005).

### Fluorescent Immunohistochemistry

Adult rat offspring (70 day old) of dams given either LPS or pyrogen-free saline in the presence or the absence of IL-6Ab on GD15 were anesthetized with urethane (1.5 g/kg, i.p.), transcardially perfused with ice cold saline followed by a 10% neutral-buffered formalin solution. The rat brains were removed from the skull and post-fixed for at least 2 consecutive days. They were subsequently embedded in paraffin and processed for immunofluorescent staining as previously described (Mouihate, 2014). Serial thin coronal sections (5 μm) of paraffin embedded brains were performed through the rostral, medial and caudal parts of the SVZ at interaural locations of ∼10.5, 9.5, and 8.5, respectively, according to the rat stereotaxic coordinates (Paxinos and Watson, 2005) and mounted on super-frost plus slides (VWR international, Arlington Heights, IL, USA). Brains were also serially cut through rostral, medial and caudal parts of the DG at interaural locations of ∼6.5, 5.5, and 4.5, respectively. Brain sections were incubated with either doublecortin (DCX) antibody made in goat (1:1000, Santa Cruz biotechnology,Paso Robles, CA, USA) or T-box brain protein 2 (Tbr2) antibody made in chicken (Tbr2) (1:1000, EMD Millipore, MA, USA) followed by Alexa Fluor 488 bound secondary antibodies (donkey anti-goat IgG for DCX, or donkey anti-chicken for Tbr2; 1:1000; Life Technologies, La Jolla, CA, USA). DCX expression was used to monitor ongoing neurogenesis (Rao and Shetty, 2004; Couillard-Despres et al., 2005), while Tbr2 was used to monitor intermediate progenitor cells prior to their commitment to neuronal phenotype (Hodge and Hevner, 2011). Tbr2 protein is indispensable for neurogenesis in the DG of adult rodents (Hodge et al., 2012). To assess the long term effect of LPS on microglial activation and their potential negative impact on neurogenesis, microglial cells were immunostained using an antibody anti-ionized calcium-binding adapter molecule 1 (Iba1) made in rabbit (1:1000, Wako Chemicals, Inc., Richmond, VA, USA) followed by an Alexa Fluor 555 tagged secondary antibody (1:1000, Life Technologies, La Jolla, CA, USA) as previously described (Mouihate, 2014). All antibodies used in the present paper had been previously validated. Omission of the primary antibodies led to an absence of fluorescent signal.

Three coronal sections at each of the rostral, medial and caudal regions of either the SVZ or the DG of male rat offspring were monitored for cell count. Each rat group contains cell count from 4 to 7 of male rats born to different dams given either saline or LPS in the presence or the absence of IL-6Ab injection. The cell count is derived from a total of 9 brain sections per rat in each neurogenic area (SVZ or DG). The DCX^+^ and Tbr2^+^ cells in both left and right of SVZ and DG areas of the brain were counted by an experimenter blind to the rat’s treatment using a 20x objective (Axio imager A1, Carl Zeiss Microscopy GmbH, Germany). Iba1^+^ were counted in the hilus of the hippocampus and expressed as number of cells per area (in mm^2^). The number of Iba1^+^ along the granular cell layer (GCL) of the DG were counted. A line was drawn at the base of the GCL, its length was measured and the data were presented as the number of Iba1^+^ per line length (in mm). The area of the hilus and length of GCL base were measured using imageJ software (Schneider et al., 2012). All immunofluorescent images presented in this paper were acquired using a confocal laser scanning microscope (Carl Zeiss Microscopy GmbH/Germany).

### Administration of RU486

In a different series of experiments, male rats born to dams given either saline or LPS during pregnancy were injected with LPS (1 mg/kg, i.p.) during adulthood. These rats were given either 50 mg/kg (i.p.) of the glucocorticoid receptor antagonist RU486 (Sigma–Aldrich) dissolved in dimethyl sulfoxide (DMSO) or DMSO alone as previously described (Ellis et al., 2005). Twenty four hours later, rats were transcardially perfused with ice cold saline and brains collected and processed for immunofluorescent detection of DCX and Tbr2 as described above. Another cohort of adult rats were given saline instead of LPS and their brain were processed for immunofluorescent detection of Tbr2.

### Data Analysis

All data were compared using 2-way ANOVA followed by Student–Newman–Keuls *post hoc* test whenever possible. The difference between groups was declared statistically significant at *p* < 0.05. Results are shown as mean ± SEM.

## Results

**FIGURE 1:**
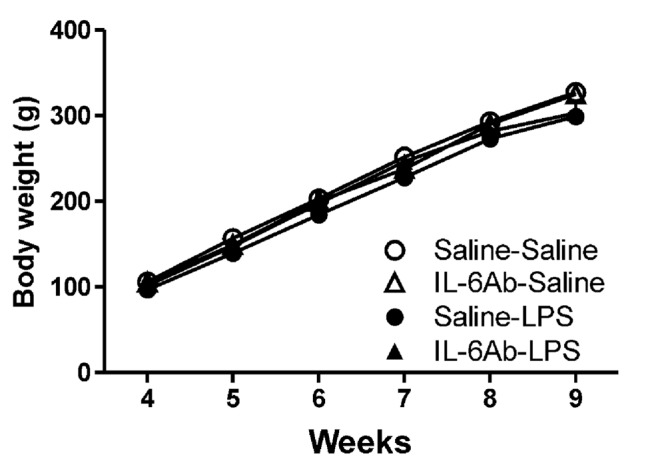
**Prenatal lipopolysaccharide (LPS) does not affect body weight gain.** Pregnant dams were injected with either LPS (100 μg/kg, i.p.) or pyrogen-free saline (equi-volume) in the absence or the presence of IL-6Ab (10 μg/kg, i.p.) on gestation day 15. One male offspring from each dam was selected randomly and its weight was monitored weekly until postnatal week 9. Note that neither prenatal LPS nor prenatal IL-6Ab did affect the body weight gain of adult male offspring (Saline–Saline: *n* = 5, IL-6Ab–Saline: *n* = 7, Saline–LPS: *n* = 5, IL-6Ab–LPS: *n* = 5).

**Figure 1** shows that body weight gains in rats born to dams injected with pyrogen-free saline or LPS in the absence or the presence of IL-6Ab. Injection of either LPS (100 μg/kg, i.p.) or IL-6Ab during pregnancy did not affect the offspring’s body weight gain. Previous study has also shown that prenatal LPS or IL-6Ab does not affect the litter size (Mouihate and Mehdawi, 2016). It appears that this injection regimen does not result in overt growth restriction or growth delay.

**FIGURE 2:**
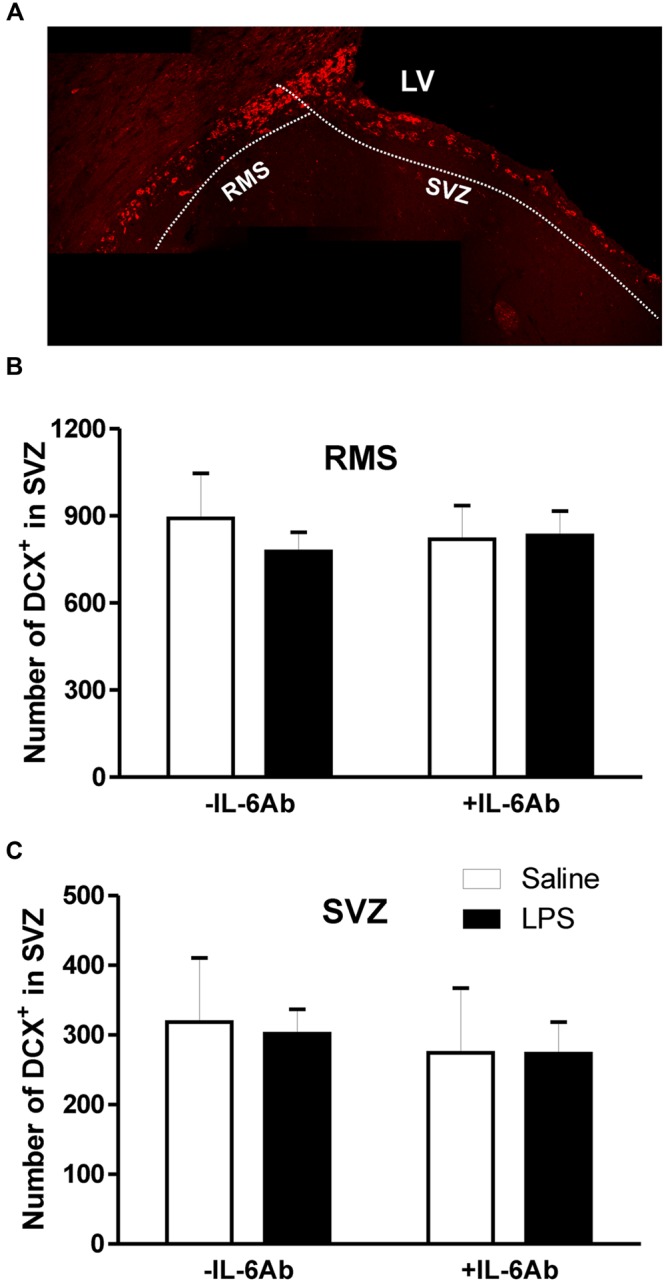
**Prenatal LPS does not affect the number of newly born neurons in the subventricular zone.** Pregnant dams were injected with either LPS (100 μg/kg, i.p.) or pyrogen-free saline (equi-volume) in the absence or the presence of IL-6Ab (10 μg/kg, i.p.) on gestation day 15. Newly born neurons were counted in the subventricular *proper* and the rostral migratory stream of adult male offspring. Micrograph in **(A)** shows typical doublecortin staining the subventricular zone. In the absence of IL-6Ab (-IL-6Ab), prenatal LPS did not significantly affect the number of doublecortin cells in either the rostral migratory stream **(B)** or the subventricular zone **(C)**. Such lack of prenatal LPS impact on adult neurogenesis were also observed when LPS and IL-6Ab (+IL-6Ab) were co-administered to pregnant dams (Saline–Saline: *n* = 5, IL-6Ab–Saline: *n* = 5, Saline–LPS: *n* = 6, IL-6Ab–LPS: *n* = 5).

Because DCX expression in adult brains reflects neurogenesis (Couillard-Despres et al., 2005), we used this marker to monitor newly born neurons in the SVZ and DG. As shown in **Figure 2**, DCX cells were counted separately in two sub-regions, the SVZ proper and in the rostral migratory stream area (RMS). Administration of LPS to pregnant dams did not result in any significant effect on the number of DCX containing cells in either the SVZ or the RMS of adult offspring. Similarly, there was no significant effect of prenatal administration of IL-6Ab on the number of DCX containing cells in either areas.

**FIGURE 3:**
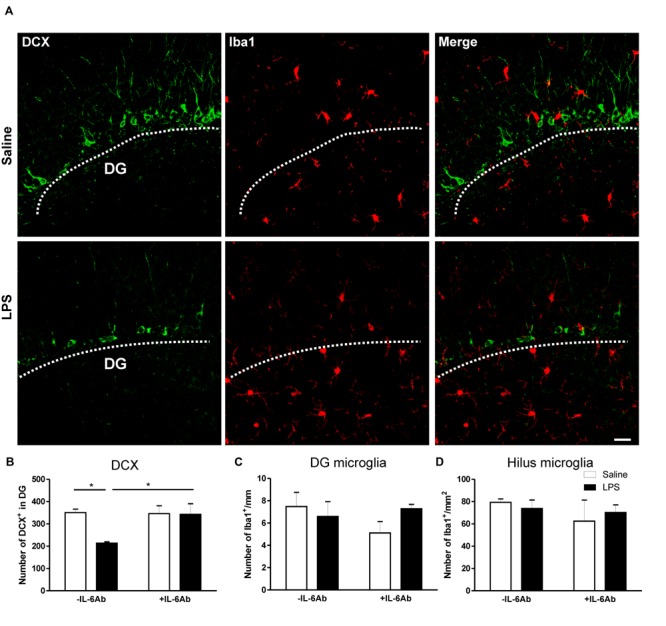
**Prenatal LPS reduces the number of newly born neurons in the dentate gyrus of the hippocampus.** Pregnant dams were injected with either LPS (100 μg/kg, i.p.) or pyrogen-free saline (equi-volume) in the absence or the presence of IL-6Ab (10 μg/kg, i.p.) on gestation day 15. Newly born neurons were counted in the dentate gyrus of the hippocampus. Micrographs in **(A)** show immunofluorescent staining of doublecortin (green: left column), microglia (red: middle column) and combined staining of doublecortin and microglia (right column) in the absence (Saline: upper row) or the presence of prenatal LPS injection (LPS: lower row). The graph bars in **(B)** show the number of doublecortin containing cells in the dentate gyrus of adult male offspring. In the absence of the IL-6Ab (-IL-6Ab), LPS injection to pregnant dams (solid bars) resulted in a significant decrease in the number of doublecortin containing cells in the dentate gyrus of adult male offspring when compared to those born to dams given saline only (open bars). Such long lasting impact of prenatal LPS was absent when IL-6Ab was co-administered with LPS injection during gestation period (+IL-6Ab). The graph bars in **(C)** and **(D)** show the count of microglial cells in the dentate gyrus **(C)** and the hilus **(D)** of the hippocampus of adult offspring born to dams given either saline (open bars) or LPS (solid bars) in the absence (-IL-6Ab) or the presence of IL-6Ab (+IL-6Ab) during pregnancy. Note that the number of microglia was not significantly altered by prenatal administration of LPS and/or IL-6Ab (Saline–Saline: *n* = 5, IL-6Ab–Saline: *n* = 7, Saline–LPS: *n* = 5, IL-6Ab–LPS: *n* = 5). DG = Dentate gyrus. ^∗^*p* < 0.05. Scale bar = 50 μm.

In contrast, prenatal administration of LPS led to a reduced number of DCX containing cells in the DG of the hippocampus (micrograph in **Figure 3**). Interestingly, co-administration of IL-6Ab and LPS during pregnancy halted this prenatal LPS-induced reduction in the number of newly born neurons in adults (**Figure 3B**). Experimental evidence suggests that prenatal immune challenge dampens neurogenesis was associated with enhanced microglial activation (Graciarena et al., 2010, 2013). Therefore, we assessed whether prenatal LPS had an effect on microglial activation in the hippocampal area. Activated microglia are characterized by enlarged perikaryon, shorter processes and increased mitotic activity (Mouihate, 2014). In the present study, we did observe no obvious morphological changes of microglia in response to prenatal administration of LPS in the presence or the absence of IL-6Ab. In addition, prenatal administration of LPS in the presence or the absence of IL-6Ab did not alter the number of microglia in either the hilus of the hippocampus (**Figure 3C**) or along the basal area of the DG (**Figure 3D**).

**FIGURE 4:**
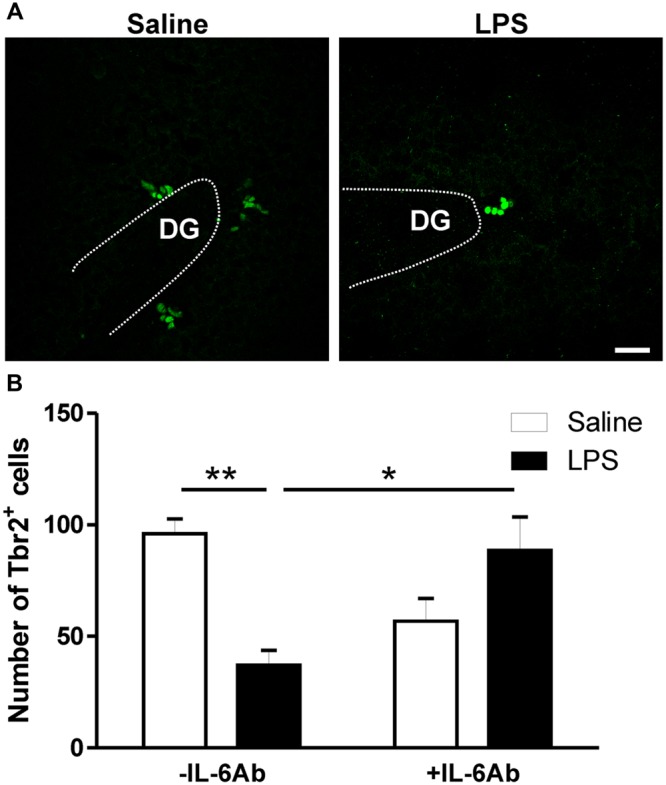
**Prenatal LPS reduces the number of intermediate neuronal progenitors in the dentate gyrus of the hippocampus.** Pregnant dams were injected with either LPS (100 μg/kg, i.p.) or pyrogen-free saline (equi-volume) in the absence or the presence of IL-6Ab (10 μg/kg, i.p.) on gestation day 15. Intermediate neuronal progenitor cells expressing T-box brain protein 2 (Tbr2) were counted in the dentate gyrus of the hippocampus. Micrographs in **(A)** show immunofluorescent staining of Tbr2 in male adult offspring born to dams given either saline (left panel) or LPS (right panel). The graph bars in **(B)** show the number of Tbr2 containing cells in the dentate gyrus of adult male offspring. In the absence of the IL-6Ab (-IL-6Ab), LPS injection to pregnant dams (solid bars) resulted in a significant decrease in the number of Tbr2 containing cells in the dentate gyrus of adult male offspring when compared to those born to dams given saline only (open bars). Such long lasting impact of prenatal LPS was absent when IL-6Ab was co-administered with LPS injection during gestation period (+IL-6Ab) (Saline–Saline: *n* = 5, IL-6Ab–Saline: *n* = 7, Saline–LPS: *n* = 5, IL-6Ab–LPS: *n* = 5). DG = Dentate gyrus. ^∗^*p* < 0.05, ^∗∗^*p* < 0.01. Scale bar = 50 μm.

In addition to DCX labeling of newly born neurons, we also assessed whether prenatal LPS affects Tbr2 containing cells within the DG. Tbr2 is a transcription factor expressed by intermediate neuronal progenitors which are largely committed to become glutamatergic neurons and is indispensable for neurogenesis in the DG during adulthood (Hodge and Hevner, 2011; Hodge et al., 2012). Prenatal administration of LPS led to reduced number of Tbr2 containing cells in the DG of adult rats. Interestingly, such effect was lost when pregnant dams were concomitantly given the IL-6Ab (**Figure 4**).

Prenatal activation of TLR4 has been associated with increased HPA responsiveness to LPS in adult offspring (Enayati et al., 2012; Lin et al., 2012; Mouihate, 2012; Zager et al., 2014). Because glucocorticoids, the final product of HPA axis activity, negatively impacts neurogenesis (Schoenfeld and Gould, 2012), we assessed whether the programming effect of prenatal LPS on adult neurogenesis can be affected when glucocorticoid receptors (GR) are antagonized. For this reason, DCX and Tbr2 containing cells were monitored in the DG of adult offspring subsequently injected with LPS in the presence or the absence of the GR antagonist RU486.

**FIGURE 5:**
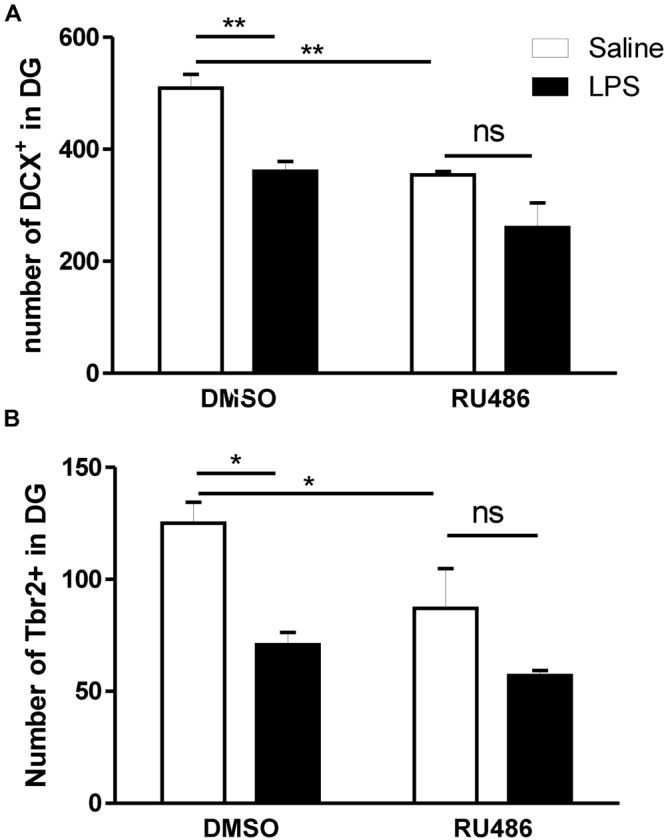
**Impact of glucocorticoid antagonist on the long lasting impact of prenatal LPS on newly born neurons in the dentate gyrus of immune challenged adult offspring.** Pregnant dams were injected with either LPS (100 μg/kg, i.p.) or pyrogen-free saline (equi-volume) on gestation day 15. Adult male offspring were subsequently injected with LPS (1 mg/kg, i.p.) in the absence [dimethyl sulfoxide (DMSO)] or the presence of the glucocorticoid receptor antagonist RU486 (dissolved in DMSO). Cell count of doublecortin **(A)** and T-box brain protein 2 (Tbr2) containing cells **(B)** was monitored in the dentate gyrus of LPS-injected adult offspring born to dams given either saline (open bars) or LPS (solid bars) on gestation day 15. In vehicle (DMSO)-treated adult offspring, rats born to dams given LPS during pregnancy and given LPS during adulthood showed a significantly decreased number of both doublecortin (DMSO: panel A) and Tbr2 (DMSO: panel B) containing cells in their dentate gyrus when compared to those born to saline-injected dams and given LPS during adulthood. The long lasting impact of prenatal LPS on newly born neurons were absent when RU486 was co-injected with LPS during adulthood (Saline–DMSO: *n* = 4, Saline–RU486: *n* = 4, LPS–DMSO: *n* = 4, LPS–RU486: *n* = 3). ^∗^*p* < 0.05, ^∗∗^*p* < 0.01.

In the absence of adult treatment with RU486 (DMSO rat group), the prenatal injection of LPS led to a significant reduction in the number of both DCX (**Figure 5A**) and Tbr2 (**Figure 5B**). However, such programing effect of LPS on neurogenesis was absent when adult offspring were given RU486.

## Discussion

The mechanisms underlying the long lasting negative impact of prenatal immune stress on adult neurogenesis is not well understood. This study is the first to show that IL-6 plays an important role in mediating the long lasting impact of prenatal LPS on adult neurogenesis. The data presented here complement our previous observation on the potential direct impact of LPS-induced mobilization of maternal IL-6 on fetal brain and its consequent contribution to the long lasting impact of prenatal LPS (Mouihate and Mehdawi, 2016).

Activation of maternal innate immune system results in increased synthesis and secretion of a plethora of pro-inflammatory cytokines, the most important of which are TNFα, IL-1β, and IL-6 (Mouihate et al., 2005; Boksa, 2010). Several studies suggest that IL-6, but not IL-1β or TNFα, can potentially cross the placenta and directly act on the fetal brain (Urakubo et al., 2001; Zaretsky et al., 2004; Dahlgren et al., 2006; Hsiao and Patterson, 2011; Mouihate and Mehdawi, 2016). Thus, LPS-induced IL-6 could likely affect the developmental trajectory of the fetal brain.

There are conflicting data as to whether IL-6 promotes or dampens neurogenesis in adulthood. For example a chronic transgenic expression of IL-6 by astrocytes dampens hippocampal neurogenesis (Vallieres et al., 2002), while a complete absence of the IL-6 gene in an IL-6 KO mice model led to rather a decrease in neurogenesis (Bowen et al., 2011). The chronic loss or gain of function of IL-6 gene are likely associated with compensatory mechanisms that are difficult to monitor. However, a large body of evidence suggest that prenatal exposure to bacterial or viral pathogens alters brain function and plasticity (Boksa, 2010). Hence, this study was designed to test the potential role of IL-6 in bacterially driven activation of the maternal innate immune response and its long lasting impact on neurogenesis in adult offspring. We have recently shown that the injection of the IL-6Ab to pregnant dams reduced LPS-induced IL-6 in their plasma and dampened phosphorylation levels of the signal transducer and activator of transcription-3 (STAT-3) in the brain of their fetuses (Mouihate and Mehdawi, 2016). In the present study, we explored whether such neutralizing effect of IL-6Ab reverted the long lasting impact of LPS on adult neurogenesis. Our data show that immune-based neutralization of the inflammatory cytokine IL-6 during pregnancy halted the long lasting negative impact of TLR4 activation on neurogenesis in adult offspring. Whether such recovered neurogenesis is associated with unaltered learning and memory is still unclear. In addition to TLR4, activation of maternal TLR3, a viral receptor, resulted in altered behaviors including schizophrenia-like behaviors, deficit in exploratory and social behavior. These symptoms were absent in mice devoid of *IL-6* gene or when the pregnant dams were given an IL-6 neutralizing antibody (Smith et al., 2007). Thus, it appears that IL-6 plays an important role in the long lasting negative impact of both viral and bacterial infection during prenatal period. However, the mechanism(s) underlying the long lasting impact of IL-6 is unclear. There are indications that maternal immune stress alters brain plasticity through a lasting epigenetic modification (Basil et al., 2014). Furthermore, *in vitro* studies have shown that IL-6 regulates DNA methylation in human derived cell lines (Hodge et al., 2001). Further studies are needed to determine whether maternal IL-6 is involved in the epigenetic modification of genes involved in cell death/cell survival programs within the central nervous system.

One of the most explored mechanism underlying the long lasting impact of maternal immune activation on offspring’s brain function and plasticity is the HPA axis activation. It is plausible that IL-6 mediated activation of fetal brain during pregnancy alters the developmental trajectory of the HPA axis and thus alters the HPA “set point” *in utero* (Altman and Bayer, 1986). Indeed, prenatal exposure to pathogens reprograms several brain related functions and leads to such disturbances as schizophrenia-like behavior, propensity to develop anxiety, reduced learning and memory capabilities (Khandaker et al., 2013; Wischhof et al., 2015). Several of these brain-related illnesses are associated with enhanced HPA activity and altered neurogenesis in the brain of adult offspring (Graciarena et al., 2010, 2013; Lin et al., 2012; Depino, 2015). This reduced neurogenesis could be attributed to the increased HPA axis activity and the consequent increase in glucocorticoids. We and others have shown that maternal exposure to LPS does not result in increased corticosterone levels in the plasma of adult offspring in basal condition (Lin et al., 2012; Mouihate, 2012). This observation suggests that the HPA axis activity plays a minor role in the observed depressed neurogenesis in non-immune or behaviorally stressed rats during adulthood. Because a subsequent challenge with LPS leads to heightened HPA response in adult rat born to immune challenged dams during pregnancy (Lin et al., 2012; Mouihate, 2012), we assessed whether this heightened HPA axis activity mediates the reduced neurogenesis. We observed that the long lasting impact of LPS on neurogenesis was absent when these adult rat offspring were given a glucocorticoid receptor blocker. These data suggest that the re-activated HPA axis (with either immune or behavioral challenges) could contribute to the reduction in adult neurogenesis. We have noticed that the number of newly born neurons (DCX^+^ and Tbr2^+^) was relatively higher in the experiment involving DMSO injection during adulthood. This peculiar observation hold true in rats that were either saline-injected (**Supplementary Data S1**) or immune-challenged (**Figure 5**) during adulthood. It is possible that the anti-inflammatory/neuroprotective effect of DMSO contributes to this enhanced neurogenesis (Shimizu et al., 1997; Nagel et al., 2007; Jacob and de la Torre, 2009).

We observed that prenatal immune challenge resulted in a specific alteration of neurogenesis in the DG, but not in the SVZ. This observation is akin to previous studies (Graciarena et al., 2010) and supports the notion of differential sensitivity of the SVZ and DG to maternal innate immune response to pathogens. It is noteworthy that ablation of *Tbr2* gene resulted in abolished neurogenesis in the DG during adulthood but not in the sub-ependymal zone at the lateral ventricles (Arnold et al., 2008). It is possible that neurogenesis processes in the DG and SVZ of adult offspring are under the control of different transcription factors which could be differentially affected by prenatal immune challenge.

Experimental evidence suggests that prenatal exposure to LPS induces a long lasting activation of microglia within the hippocampus (Graciarena et al., 2010, 2013), which could potentially release a series of pro-inflammatory cytokines and hamper the survival of newly born neurons (Opendak and Gould, 2015). In our hand, neither the number of microglial cells within the hippocampus of adult offspring nor their morphology appear to be affected by prenatal immune challenge. This apparent discrepancy could be due to the regimen and dose of LPS injection during pregnancy. While we gave a single injection of a febrile dose of LPS (100 μg/kg, i.p.), Graciarena and collaborators subjected the pregnant rats to 4 injections of a relatively high dose of LPS (500 μg/kg, s.c.) (Graciarena et al., 2010).

## Summary

This study gives strong evidence for IL-6 involvement in prenatal LPS induced depressed neurogenesis in adult offspring. While the heightened HPA axis activity could, at least in part, explain the reduced neurogenesis in immune or behaviorally stressed adult offspring, the reduced neurogenesis in basal condition is less likely related to the HPA axis activity. The present study opens new research avenues for exploring the long lasting impact of maternal IL-6 on transcriptional genes involved in brain plasticity of adult offspring.

## Author Contributions

AM designed the research, performed research, analyzed data, and wrote the manuscript.

## Conflict of Interest Statement

The author declares that the research was conducted in the absence of any commercial or financial relationships that could be construed as a potential conflict of interest.

## Supplementary Material

DATA S1 **Impact of glucocorticoid receptor antagonist on the long lasting impact of prenatal LPS on newly born neurons in the dentate gyrus of non-immune challenged adult offspring.** Pregnant dams were injected with either LPS (100 μg/kg, i.p.) or pyrogen-free saline (equi-volume) on gestation day 15. Adult male offspring were subsequently injected with saline in the absence (DMSO) or the presence of the glucocorticoid receptor antagonist RU486 (dissolved in DMSO). Cell count of T-box brain protein 2 (Tbr2)-containing cells was monitored in the dentate gyrus of saline-injected adult offspring born to dams given either saline (open bars) or LPS (solid bars) on gestation day 15. In vehicle (DMSO)-treated adult offspring, rats born to dams given LPS during pregnancy and given saline during adulthood showed a significantly decreased number of Tbr2 containing cells in their dentate gyrus when compared to those born to saline-injected dams. The long lasting impact of prenatal LPS on newly born neurons was absent when RU486 was co-injected with saline during adulthood. (Saline-DMSO: *n* = 4, Saline-RU486: *n* = 5, LPS-DMSO: *n* = 5, LPS- RU486: *n* = 5). ^∗^*p* < 0.05, ^∗∗∗^*p* < 0.001.
